# Congenital/primary clival meningocele: A systematic review and surgical approach based on a new proposed classification

**DOI:** 10.1007/s10143-026-04435-2

**Published:** 2026-08-07

**Authors:** Pedro Henrique Costa Ferreira-Pinto, Domênica Baroni Coelho de Oliveira Ferreira, Elington Lannes Simões, Eduardo Corrêa, Othavio Gomes Lopes, Marco Aurélio Rodrigues da Fonseca Passos, Flávio Nigri

**Affiliations:** 1 Department of Surgical Specialties, Hospital Universitário Pedro Ernesto, Universidade do Estado do Rio de Janeiro, Rio de Janeiro, Brazil; 2 Department of Anatomy, Instituto de Biologia Roberto Alcântara, Universidade do Estado do Rio de Janeiro, Rio de Janeiro, Brazil

**Keywords:** Clival meningocele, Encephalocele, Primary, Congenital, Endoscopy, Clivus

## Abstract

Skull base meningoceles are extremely rare, most commonly resulting from congenital defects in sphenoid bone fusion. Inadequate embryonic fusion between the posterior sphenoid and the occipital bones may result in a transclival meningocele. Given the absence of a formal classification system for clival meningoceles and clear guidelines for the most appropriate endoscopic skull base surgical approach, we combined an anatomical study and a systematic review to characterize anatomical patterns, propose a preliminary classification, and discuss transoral and endonasal surgical corridors according to each clival subtype. In addition, we report the first known case of a primary clival meningocele successfully treated via a transoral endoscopic approach. The study was registered with PROSPERO (CRD42024575282) and received approval from the Ethics Committee (82878724.1.0000.5259). The systematic review followed PRISMA guidelines. PubMed/MEDLINE, Embase, and Cochrane were rigorously searched. Anatomical dissection of midsagittal skull base specimens was performed to assess surgical access to clival regions. Seventeen studies comprising 18 patients (mean age 30.7 years; 72.2% female) were included. Clival defects were classified as posterior transsphenoidal (n = 11), spheno-occipital (n = 1), and basioccipital (n = 6). The endoscopic endonasal approach appeared more appropriate for posterior transsphenoidal cases and the transoral route for basioccipital defects. Anatomical dissection studies confirmed that the endonasal corridor is more suitable for the upper third of the clivus and the transoral route for the lower third.

## Introduction

Cranial dysraphism refers to a group of malformations characterized by skull and dural defects with extracranial herniation of the leptomeninges, brain, and cerebrospinal fluid (CSF) [44, 48]. They are classified according to a) etiology: primary (or congenital), secondary (or acquired), combined (multifactorial) or idiopathic [2, 38, 49, 52]; b) anatomical site: occipital, convexity, sincipital, or basal [48, 50]; and c) contents: meningocele, consisting of meninges and CSF [48]; and meningoencephalocele (or encephalocele/cephalocele), including meninges, CSF, and neural tissue [48]. The terms “meningocele” and "encephalocele" have been used as synonyms, regardless of the presence or absence of neural tissue [18].

Basal encephalocele is the least common type, with an incidence of about 1 in 35,000 live births [25]. In a large series reported by Ingraham et al. (1943) [18], out of 84 patients with cranial encephaloceles, only six (approximately 7%) were basal and only one (1.2%) involved a nasopharyngeal defect. Basal encephalocele is anatomically subclassified into transethmoidal, sphenoethmoidal, transsphenoidal, sphenopharyngeal, spheno-orbital, sphenomaxillary, and basioccipital [33, 34, 50]. The clivus is a shallow depression located behind the dorsum sellae, formed by the posterior portion of the sphenoid bone along with the basilar part of the occipital bone. Improper fusion or malformation of these structures during embryological development may result in a specific type of basal encephalocele: a primary clival meningocele. Only a few cases have been reported in the literature [33]. Although transoral or endonasal routes have been employed to repair these skull base defects, there is no consensus regarding the best approach.

The objective of this study is to systematically review the literature and propose a preliminary classification for clival meningoceles to facilitate surgical decision-making in the management of these complex skull base defects. An illustrative case of the first known primary clival meningocele successfully treated via the transoral endoscopic route is also included.

## Materials and methods

### Systematic review

The protocol for our systematic review was registered with the International Prospective Register of Systematic Reviews (PROSPERO) under identification number **CRD42024575282**. Furthermore, it was conducted in accordance with the Preferred Reporting Items for Systematic Reviews and Meta-Analyses (PRISMA) statement [37].

#### Inclusion and exclusion criteria

We included studies that: 1) were prospective or retrospective; and 2) reported primary or congenital clival meningoceles. There were no restrictions on publication date.

Studies that did not meet the inclusion criteria were excluded. Additional exclusion criteria were: 1) clival defects secondary to trauma, infection, metabolic disease, tumors, idiopathic intracranial hypertension, and multifactorial defects; 2) clival meningocele or CSF leak without a clear etiology; 3) occult clival defects, including canalis basilaris medianus (CBM), the craniopharyngeal canal, and fossa navicularis magna; 4) meningocele without an identifiable clival anatomical defect; 5) CSF leak without meningocele; 6) meningocele or clival encephalocele without sufficient clinical details or specifications; 7) papers in languages other than English, Spanish, or Portuguese; 8) systematic reviews, meta-analyses, and editorial letters; and 9) duplicate data. In cases of duplicate data, only the study with the largest number of patients or the one that contained more variables of interest was included.

#### Search strategy

PubMed/MEDLINE, Embase, and Cochrane were systematically searched in March 2024 with the following strategy: ("clival" OR "clivus" OR "transclival") AND ("meningocele" OR "encephalocele" OR "meningoencephalocele" OR "cephalocele"). The reference lists of included articles were manually reviewed for additional relevant publications. An updated literature search was conducted on April 28th, 2025 to identify newly eligible studies.

#### Data Extraction, synthesis, and assessment of risk of bias

Data were independently extracted by two reviewers (DBCOF and PHCFP) and included author details, year of publication, study design, sample size, patient demographics, clinical presentation, treatment modalities, and surgical details or complications. RStudio [43] was used to summarize patient demographics and clinical presentations. Publication bias was assessed using the Joanna Briggs Institute (JBI) Critical Appraisal Checklist for Case Reports and Case Series tools [13, 35], and the robvis tool [30] was used for visualization. Disagreements were resolved through consultation with a third senior reviewer (FN).

### Anatomy

Anatomical dissections were performed on five formalin-fixed specimens consisting of midface sagittal head sections without obvious intracranial pathology at the Human Anatomy Laboratory. A 4 mm-diameter, 18 cm-long rigid endoscope (Karl Storz Gmbh, Tuttlingen, Germany), connected via a fiber-optic cable to a high-performance light source and equipped with a 3-charge-coupled device camera (Stryker, USA), was used for dissection. The clival approach via the endonasal and transoral routes was evaluated, focusing on the anatomical relationships between the clivus, the nasal and oral cavities, and the hard and soft palates.

## Results

### Systematic review

#### Study selection

**Fig. 1:**
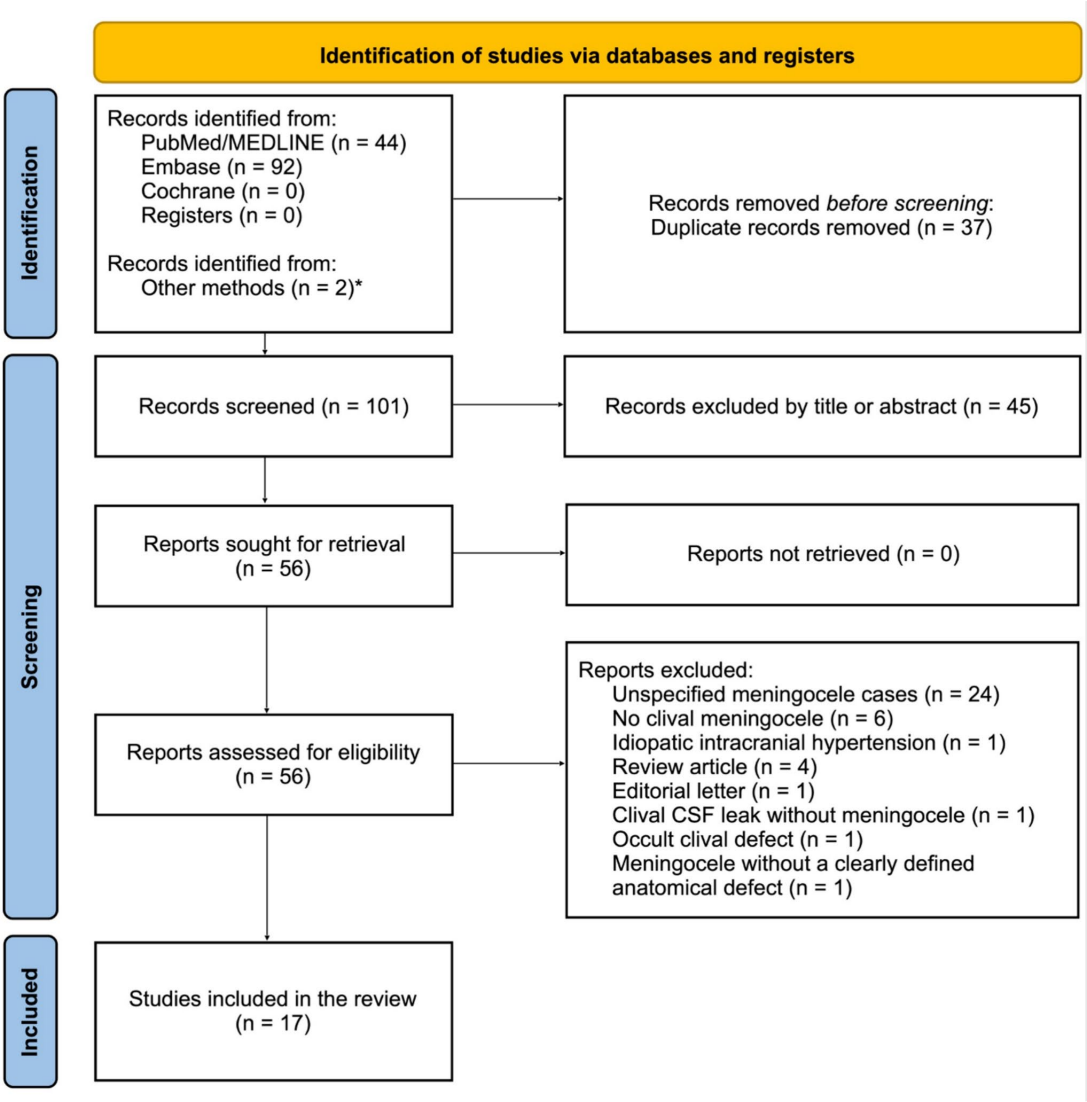
PRISMA flow diagram of the systematic search of primary clival meningocele. *Other methods refer to manual searches of reference lists

As detailed in Fig. 1, the initial search yielded 136 manuscripts. After removing duplicates and ineligible studies, 54 remained and were fully assessed based on the inclusion criteria. Of these, 15 were included. Following a careful review of their reference lists, two additional studies were identified. In total, we selected 17 articles, comprising 18 patients with primary clival meningocele.

#### Pooled analysis

Eighteen cases were identified and summarized in Tables 1 and 2, according to the location of the defect in the clivus (posterior transsphenoidal, spheno-occipital, and basioccipital). The mean age was 30.7 ± 29.9 years, ranging from neonates to 78 years old. Only 33.3% of patients presented with associated malformations, mostly in the basioccipital group. Within this group, Ko (2010) [24] detailed a patient with a hypoplastic C1 ring, hemivertebrae of C2 and C3, and curvature of the upper cervical spine to the left; Manjila (2012) [28] documented duplication of the pituitary gland, cleft lip and palate, duplication of the basilar artery, a nasopharyngeal teratoma, an absent corpus callosum, a bifid odontoid process, thoracic scoliosis, a bifid tongue and uvula, and severe intellectual disability; Nager (1987) [36] described an arachnoid cyst at L2-L3 (L3 left root) with surgical closure at 9 and 13 months; and Puvabanditsin (2014) [41] reported hypoplasia of the cerebellum and a 5q15 deletion. In the posterior-transsphenoidal group, Kaptain (2000) [19] identified a midline fusion defect of the clivus, while in the spheno-occipital group, Sharma (2017) [46] reported a right-sided parieto-occipital and medial part of temporal bone defect, as well as a hypoplastic condyle and hypoglossal canal. Finally, the most prevalent symptoms occurred predominantly in patients with posterior transsphenoidal defects.

**Table 1:** Overview of epidemiological and clinical characteristics in cases of primary clival meningoceles

Variable	Basioccipital N = 6^1^	Posterior transsphenoidal N = 11^1^	Spheno-occipital N = 1^1^	Overall N = 18^1^
Sex				
Female	4 (66.7%)	9 (81.8%)	-	13 (72.2%)
Male	2 (33.3%)	2 (18.2%)	1 (100%)	5 (27.8%)
Location				
America	5 (83.3%)	4 (36.4%)	-	9 (50%)
Asia	-	3 (27.3%)	1 (100%)	4 (22.2%)
Europe	1 (16.7%)	3 (27.3%)	-	4 (22.2%)
Middle East	-	1 (9.1%)	-	1 (5.6%)
Age (years)^2^	2.2 (4.3)	47.5 (26.3)	17 (NA)	30.7 (29.9)
Defect length (cm)^3^				
< 5	2 (33.3%)	2 (18.2%)	-	4 (22.2%)
5–10	1 (16.7%)	1 (9.1%)	-	2 (11.1%)
10–20	1 (16.7%)	2 (18.2%)	-	3 (16.7%)
> 20	1 (16.7%)	1 (9.1%)	-	2 (11.1%)
Unknown	1 (16.7%)	5 (45.5%)	1 (100%)	7 (38.9%)
Defect content				
Meningeal tissue	2 (33.3%)	1 (9.1%)	-	3 (16.7%)
Meningeal tissue + CSF	4 (66.7%)	10 (90.9%)	1 (100%)	15 (83.3%)
Associated malformations				
Yes	4 (66.7%)	1 (9.1%)	1 (100%)	6 (33.3%)
No	2 (33.3%)	9 (81.81%)	-	11 (61.1%)
Unknown	-	1 (9.1%)	-	1 (5.5%)
Main symptoms^4^				N = 16^1^
CSF leak/rhinorrhea	-	8 (72.7%)	-	8 (50%)
Meningitis	3 (50%)	5 (45.5%)	-	8 (50%)
Respiratory Distress	3 (50%)	2 (18.2%)	-	5 (31.2%)

^1^n (%), except age (years); ^2^ Mean (SD); ^3^ For cases in which three dimensions of the cystic area were reported, the largest measurement was considered as it best guides surgical closure; ^4^ Only 16 patients present symptoms, and a single patient may exhibit more than one simultaneously.

**Table 2:** Clinical and surgical characteristics of reported cases of congenital clival meningocele

Study	Cases (n)	Clival defect location	Clinical presentation	Surgical Approach	Magnification method	Defect closure	Reoperated	Surgical complications
Azizkhan 1989 [3]	1	Basioccipital	Intrauterine: polyhydramnios, violent pharyngeal contractions At birth: cyanosis, bradycardia, stridor, increased oral secretions, and congenital right torticollis	Transoral	Microsurgery	Suture with muscle and biological adhesive	No	Impairment of pharyngeal motility in which improved over 2 years
Ko 2010 [24]	1	Basioccipital	Meningitis and sepsis	Combined endonasal and transoral	Endoscopic	Porcine intestinal submucosal graft with fibrin glue	No	No
Manjila 2012 [28]	1	Basioccipital	Incidental finding during nasopharyngeal teratoma MRI investigation	None	NA	NA	NA	NA
Nager 1987* [36]	1	Basioccipital	Nine previous episodes of bacterial meningitis	Transoral	Microsurgery	Suture with muscle and biological adhesive	No	No
Panagopoulos 2019 [38]	1	Basioccipital	Rhinitis, respiratory distress/stridor, hypotony, pallor, retropharyngeal secretions, and meningitis	Transoral	Direct view	Mass infundibulum double ligated with 2–0 silk suture, and submuscular dissection of the pharyngeal tissue around the failure	No	No
Puvabanditsin 2014* [41]	1	Basioccipital	Preterm, respiratory distress, tracheostomy and gastrostomy tube	Awaiting clinical stabilization to encephalocele repair	NA	NA	NA	NA
Akyuz 2008 [1]	1	Posterior transsphenoidal	1-year history of intermittent CSF rhinorrhea through her left nostril	Endonasal	NA	Packing of the sphenoid sinus with fascia lata, fat graft and biological glue	No	No
Becker 2010 [4]	1	Posterior transsphenoidal	NA	Endonasal	Endoscopic	Nasoseptal flap, fibrin glue and fat graft	No	NA
Kaptain 2000 [19]	1	Posterior transsphenoidal	At birth: respiratory distress 7 years later: recurrent meningitis	At birth: endonasal 7 years later: sublabial-transseptal	At birth: endoscopic 7y: NA	Aneurysm clip, abdominal fat	Yes	NA
Katori 2013 [22]	1	Posterior transsphenoidal	Incidental findings during laryngoscope. No signs or symptoms	NA	NA	NA	NA	NA
Khoueir 2021 [23]	1	Posterior transsphenoidal	CSF rhinorrhea and one previous episode of meningitis	Endonasal	Endoscopic	Pedicled nasoseptal flap, cellulose hemostatic, and gasket seal. A silastic nasal splint was inserted at the level of the left nasal septum and fixed by a 2–0 silk suture	No	NA
Lockwood 1980 [27]	1	Posterior transsphenoidal	CSF leak for 10 years and recurrent bacterial meningitis	Endonasal	NA	Gelfoam, freeze-dried dura and bone plug	No	No
Pang 2015 [40]	1	Posterior transsphenoidal	Upper airway obstruction, meningitis, and CSF rhinorrhea	NA	NA	NA	NA	NA
Turanzas 1996 [51]	1	Posterior transsphenoidal	CSF leak for 4 years	Endonasal	NA	Fat, Surgicel, and Tisucol	No	No
Van Zele 2013 [52]	2	Posterior transsphenoidal	CSF rhinorrhea and headache	Endonasal	Endoscopic	Fat, fascia and a free/nasoseptal flap	No	No
		Posterior transsphenoidal	CSF rhinorrhea					
Zanabria-Ortiz 2015 [54]	1	Posterior transsphenoidal	CSF leak and bacterial meningitis	Endonasal	Endoscopic	Abdominal fat graft, cellulose hemostatic, and fibrin glue	No	No
Sharma 2017 [46]	1	Spheno-occipital	Five years of swelling on the right side of the neck	Thecoperitoneal shunt	NA	NA	No	No

^*^The case reported by Nager (1987) [36] had been previously published by Martinez (1981) [29] and Hemphill (1982) [16], while the case described by Puvabanditsin (2014) [41] was also reported by Francois (2014) [12]. Abbreviations: CSF – cerebrospinal fluid; MRI – magnetic resonance imaging; NA – not applicable or not available

Of the 11 patients with posterior transsphenoidal meningocele, 9 underwent an endonasal approach. Six of these procedures were endoscopic [4, 19, 23, 52, 54], while in the remaining three the use of intraoperative image magnification devices was not specified [1, 27, 51]. Surgical treatment was not disclosed in two cases [22, 40]. Among the six patients with a basioccipital defect, three were treated via a transoral approach—two with microsurgery [3, 36] and one under direct view [38]. One patient underwent a combined endoscopic endonasal and transoral approach [24]. In one case, repair was postponed due to the need for clinical stabilization [41], and in another, no surgical procedure was performed [28]. Only one patient had a spheno-occipital meningocele. Due to a large and complex defect, a thecoperitoneal shunt was performed instead of direct repair [46].

Multiple strategies were employed for skull base defect reconstruction, using a wide variety of autologous and synthetic materials for defect closure, as detailed in Table 2. Postoperative CSF leakage, infections, failure to thrive, respiratory distress, and rhinorrhea were not reported. One patient developed pharyngeal motility impairment with spontaneous resolution within 2 years [3]. Most studies did not provide detailed information on postoperative follow-up duration; however, the available data indicate that follow-up ranged from 3 months to 2 years.

#### Risk of bias assessment

**Fig. 2:**
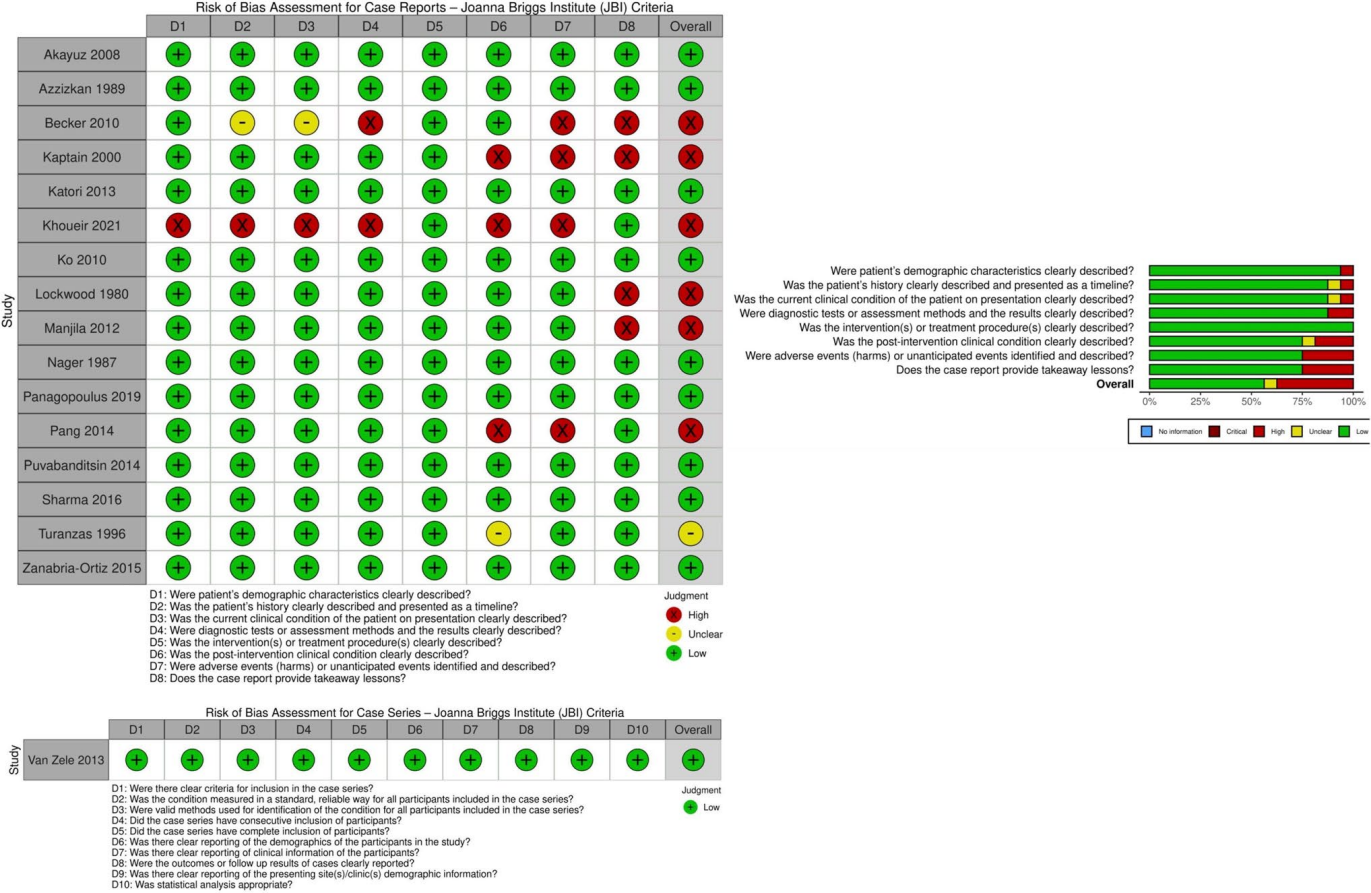
Traffic light plots and summary plot (Risk of Bias Assessment for Case Reports and Case Series—JBI Criteria)

Of the 17 included articles, 9 case reports and the case series had all the assessment items rated as low risk of bias, while one case report demonstrated a moderate risk, which indicates good methodological quality. The most consistently met criteria were D1 and D5, with low-risk judgment in all studies except one [23]. In contrast, D6, D7, and D8 showed the highest proportion of high-risk judgement. Becker (2010) [4], Kaptain (2000) [19], Khoueir (2021) [23], and Pang (2014) [40] presented the greatest number of high-risk domains, potentially affecting the reliability of their findings (Fig. 2).

As evidenced in the summary plot of case reports, five domains were positively evaluated in more than 85% of the studies, while D6, D7, and D8 demonstrated high-risk judgments in approximately 25–30% of cases. Overall, 55% of the assessed items met the criteria for low risk of bias, indicating moderate methodological quality (Fig. 2). Given the rarity of primary clival meningoceles, the overall assessment can be considered moderate to acceptable, despite the inherent limitations of case reports.

### Classification

**Fig. 3:**
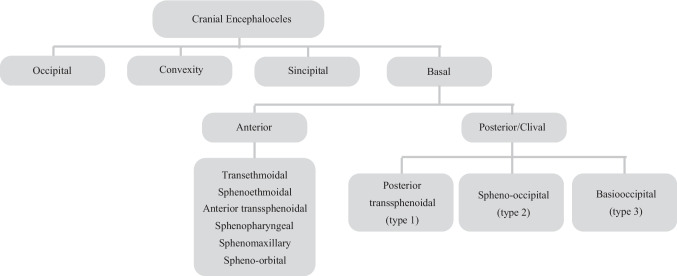
Preliminary general classification for cranial encephaloceles

**Fig. 4:**
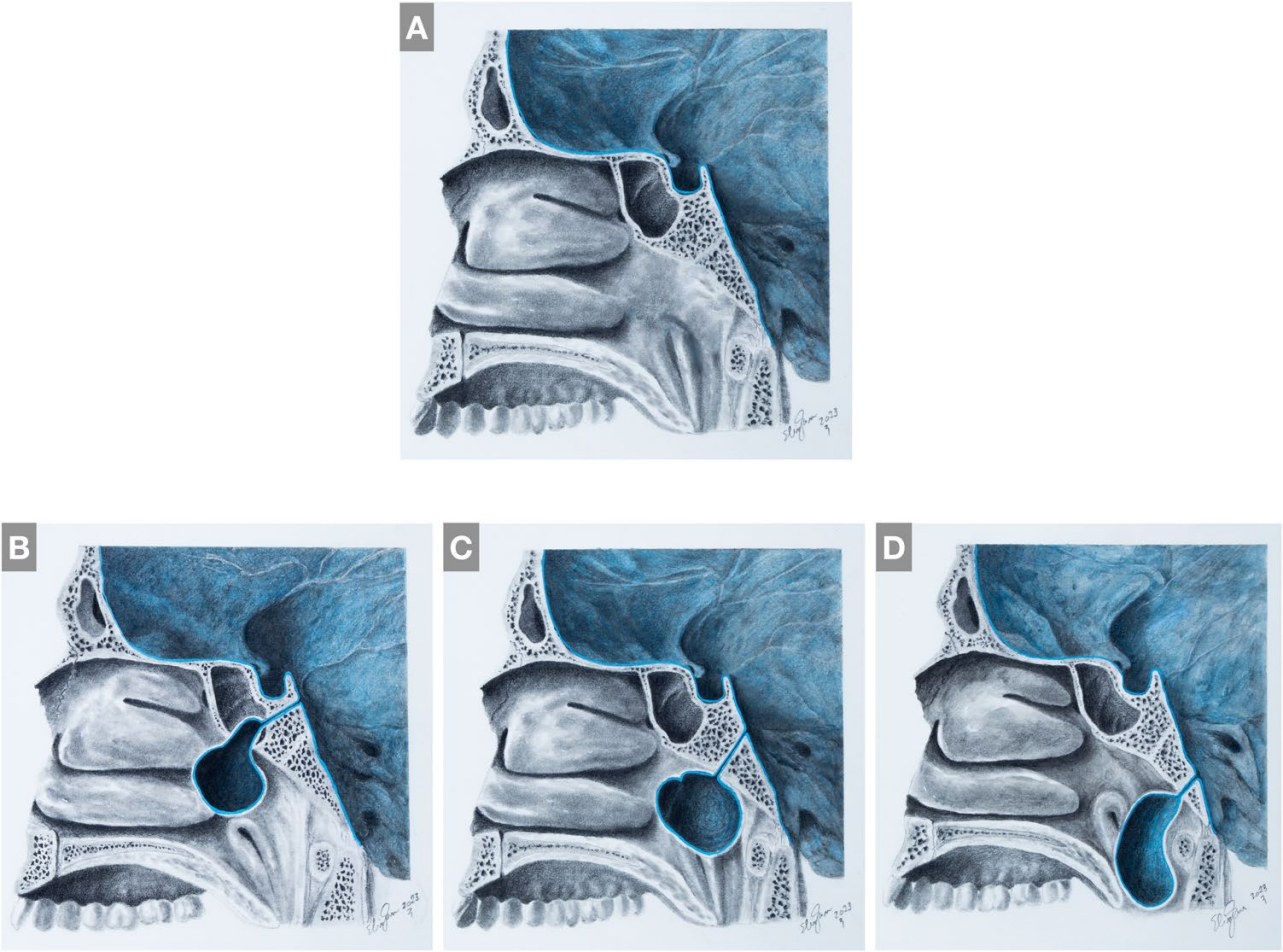
Clival meningocele classification based on site. (**A**) Normal clival anatomy without evidence of herniation. The dura mater is illustrated in blue. (**B**) Posterior transsphenoidal—Type 1: herniation through a defect in the sphenoid part of the clivus. (**C**) Spheno-occipital—Type 2: herniation through the spheno-occipital suture of the clivus. (**D**) Basioccipital—Type 3: herniation through a defect in the occipital portion of the clivus. Original illustration by co-author Dr. Elington Lannes Simões

The general anatomical classification of midline cranial encephaloceles includes occipital, convexity, sincipital, and basal subtypes [48, 50]. After careful literature review, basal meningoceles were subdivided into anterior and posterior/clival subtypes, according to their position relative to the sphenoid bone (Fig. 3). Based on radiological studies, posterior meningoceles were classified as Type I (posterior transsphenoidal), Type II (spheno-occipital), and Type III (basioccipital), according to the location of the bone defect relative to the spheno-occipital synchondrosis (Figs. 3 and 4). In patients aged 18–25 years or older, or in cases where the synchondrosis could not be identified, the clivus was divided into three equal segments. Lesions located within the upper third were classified as Type I; those situated at the junction between the upper and middle thirds as Type II; and those involving the middle or lower thirds as Type III.

### Anatomy

**Fig. 5:**
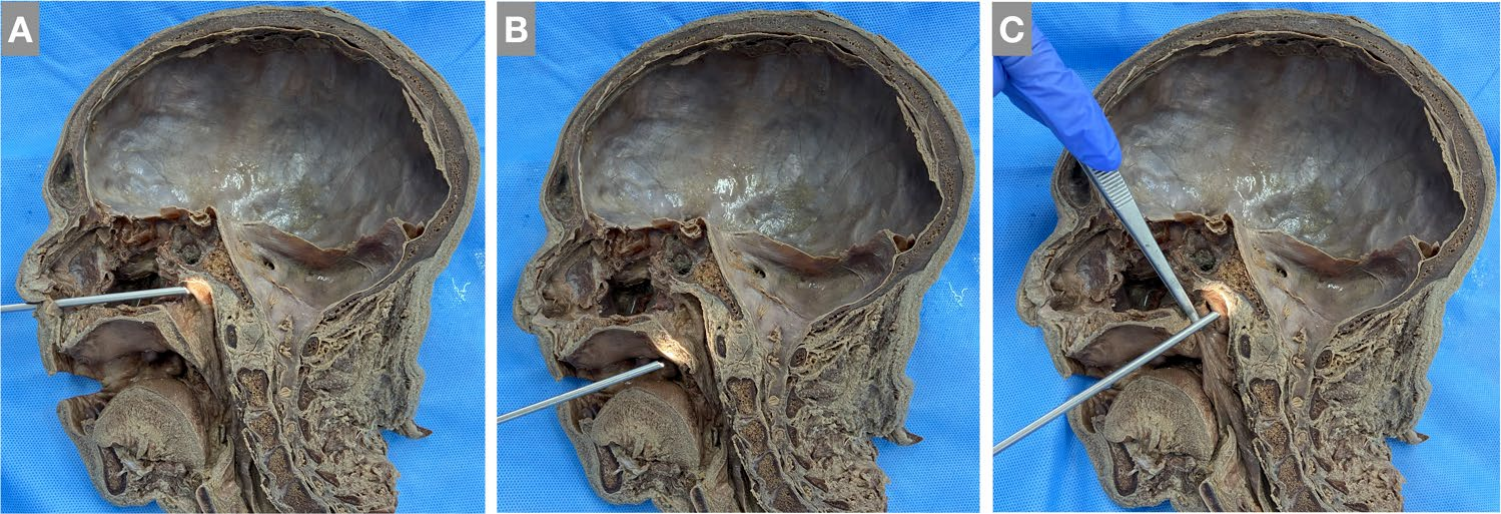
Endoscopic endonasal and transoral route evaluation in a midsagittal anatomical specimen. (**A**) Endonasal approach demonstrating access to the upper third of the clivus. (**B**, **C**) In the transoral approach, the uvula can hide the lower third of the clivus. However, with upward retraction, it is possible to have adequate exposure of this region

The midsagittal sections of five cadaveric heads were used to evaluate surgical access to the clivus. All specimens presented sellar pneumatization of the sphenoid sinus, except one with a conchal type. No clival anomalies were observed. Under high-performance illumination, the endonasal route provided a favorable surgical corridor to the upper third of the clivus, characterized by a wide field of view, optimized illumination, and a short access route, whereas the transoral approach afforded access to the lower third of the clivus based on the same qualitative criteria. These findings were consistently observed across all sections (Fig. 5).

The middle third of the clivus was a challenging region for access using conventional skull base approaches. Variables such as mouth opening, soft and hard palate anatomy, and the presence of a cleft palate were noted to hinder access to this region. Both endonasal and transoral routes were considered viable options. In two specimens with a large soft palate, superior repositioning allowed adequate access to the middle third of the clivus through the transoral approach.

### Illustrative case

A 4-month-old boy had been hospitalized since birth with respiratory failure and stridor secondary to an oropharyngeal obstruction, requiring orotracheal intubation with several unsuccessful extubation attempts. He also presented with dysphagia, breastfeeding difficulties, poor weight gain, and recurrent respiratory, urinary, and meningeal infections treated with multiple antibiotic regimens.

**Fig. 6:**
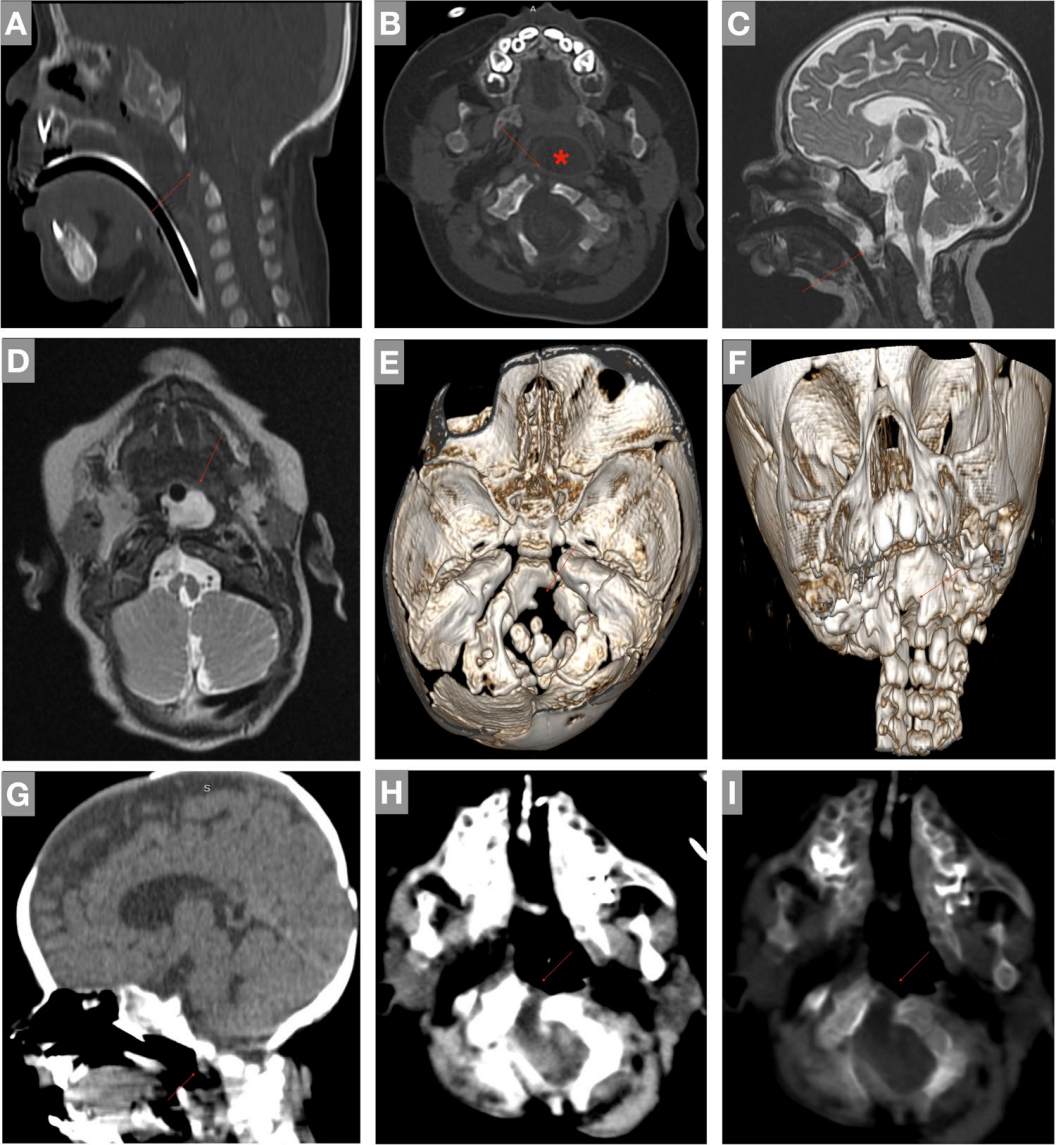
(**A**-**F**) Preoperative head CT scan and MRI images and (**G**-**I**) postoperative head CT scan. (**A**, **B**) Head CT scan—sagittal and axial bone window views showing the clival defect (red arrow) before endoscopic procedure. (**C**, **D**) T2-weighted MRI image in sagittal and axial views demonstrating the presence of the cystic lesion before the surgical procedure (red arrow). (**E**, **F**) 3D reconstruction of head CT scan showing a congenital hole in the lower third of the clivus located on the right side of the patient (red arrow). (**G**, **H**) Soft tissue window—sagittal and axial views demonstrating the absence of the cystic lesion after surgery (red arrow). (**I**) Bone window—axial view showing the clival defect (red arrow) following the endoscopic procedure, with no evidence of airway obstruction

At 2 months of age, a bronchoscopy revealed a cystic mass in the oropharynx. Subsequent head computed tomography (CT) exhibited a large cystic formation occupying the posterior nasal cavities, nasopharynx, and oropharynx, along with a clival defect. Magnetic resonance imaging (MRI) confirmed its location in the lower third of the clivus and demonstrated CSF signal intensity consistent with a meningocele (Fig. 6). A C1 anterior arch fusion defect was also identified.

**Fig. 7:**
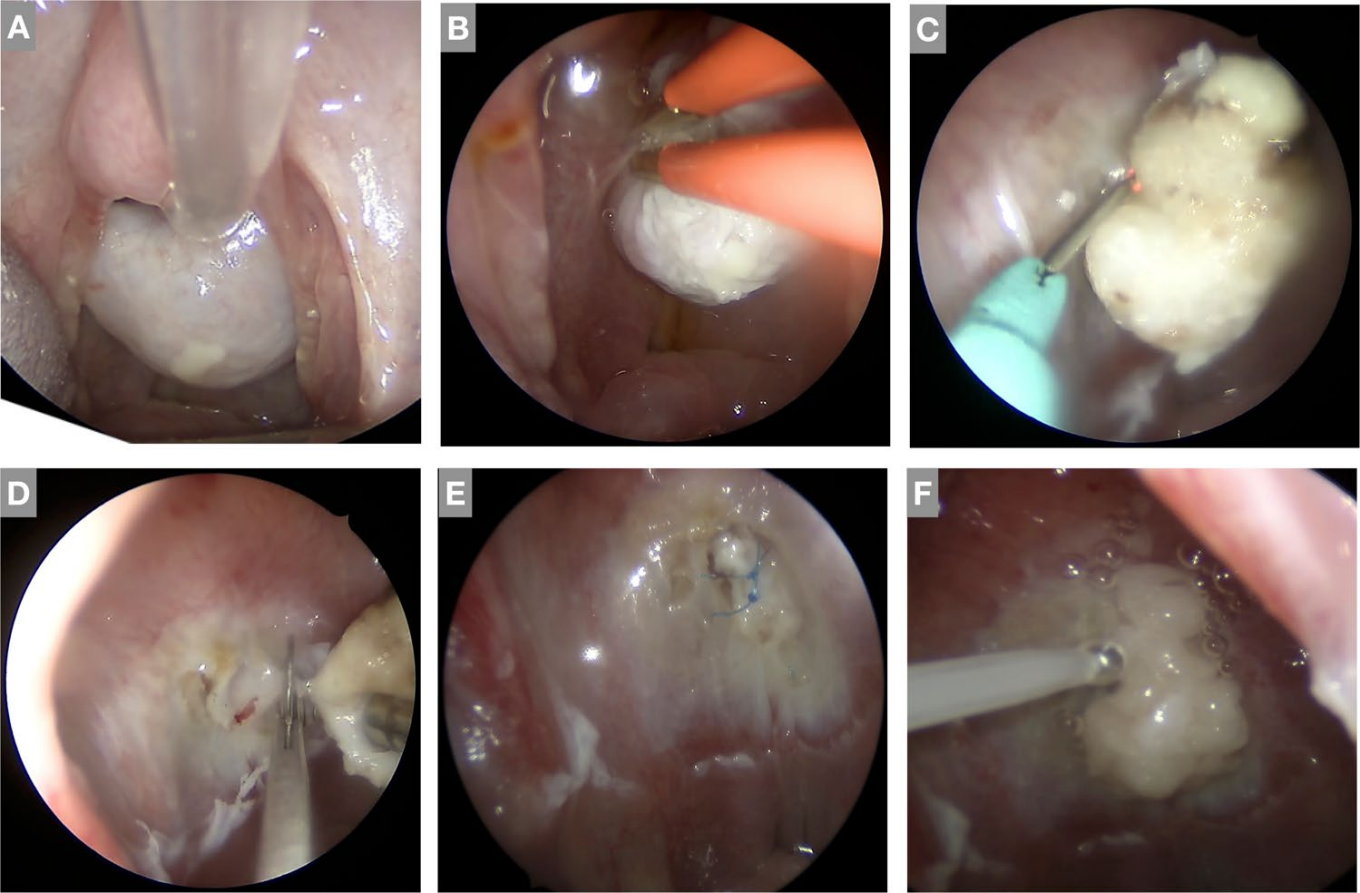
Endoscopic transoral approach for basioccipital clival meningocele. (**A**) The soft palate was retracted using a silastic band, revealing a large mass in the oropharynx. (**B**) Bipolar cautery was used to shrink the lesion. (**C**) The dural sac pedicle was resected using monopolar cautery (Colorado MicroDissection Needle). (**D**) The remaining portion of the pedicle was removed using endoscopic scissors. (**E**) The dural defect was closed with a 4–0 polypropylene suture. (**F**) Abdominal fat graft and thrombin glue were used for final reconstruction

The patient was referred to a tertiary hospital for surgical treatment at 4 months of age. The procedure was performed under general anesthesia in the supine position. An elective tracheostomy was carried out for clival manipulation and prolonged ventilatory support. A silicone catheter was inserted transnasally to retract the soft palate anteriorly. A mouth distractor and topical povidone-iodine were used for oral preparation. A 4-mm endoscope (Karl Storz GmbH & Co.) revealed a cystic pedunculated mass on the posterior wall. The lesion underwent bipolar shrinkage and was excised using monopolar cautery (Fig. 7).

After complete resection, a 5 × 5 mm defect with CSF leak was observed in the oropharynx. The meningocele was repaired with a 4–0 polypropylene suture, followed by placement of an abdominal fat graft (via Pfannenstiel incision) and fibrin glue (Fig. 7). A Valsalva maneuver confirmed the absence of residual CSF leak. No lumbar drain was placed, and there were no immediate postoperative complications. Postoperative CT scan showed total resection of the lesion (Fig. 6).

In the subsequent days, the patient developed a pulmonary infection, which responded to conservative treatment. No CSF leak was observed. He was successfully decannulated at 1 month and underwent physical and speech therapy, regaining the ability to breastfeed. At 2-year follow-up, he remained clinically stable with no evidence of recurrence or complications.

## Discussion

Encephalocele is a form of cranial dysraphism and neural tube defect characterized by herniation of brain tissue, meninges, and/or CSF. Several cases of encephalocele at various cranial sites have been reported in the medical literature. These cranial malformations result from abnormalities during brain and skull development, with defects arising during the neurulation and post-neurulation periods of human embryonic development [39].

Neurulation occurs during the third and fourth weeks of gestation after notochordal formation. During this stage, the ectoderm invaginates to form the neural tube and neural crest cells [32]. The first and classical theory suggests that incomplete closure of the cranial neuropore can cause encephaloceles. An alternative hypothesis considers a failure in the separation of the surface ectoderm from the neuroectoderm after neural fold closure. When these two layers are adherent, the paraxial mesoderm cannot interpose between them, impairing the formation of adequate skull bone and meninges [5, 17, 20, 26, 48, 50]. However, both theories fail to explain the origin of meningeal tissue, which arises from mesenchymal cells involving the encephalon. Considering these findings, some authors have started focusing on the post-neurulation development of the mesenchyme surrounding the formed neural tube as a key factor in the pathogenesis of encephaloceles [9].

The entire skull ossification during the post-neurulation stage is complex and may vary according to cranial sites. In general, bone formation occurs through the differentiation of mesenchymal cells. The mesenchyme originates from neural crest cells in the anterior skull and from mesoderm/somites in the posterior portion [10]. Some authors agree that the sphenoid line at the skull base and coronal suture in the cranial vault represent the boundaries of these cells [10, 42].

There are two types of ossification involved in the formation of the neurocranium. The cranial vault is formed through intramembranous ossification, a process in which mesenchymal cells differentiate directly into bone [45]. Controversially, the skull base and the craniocervical junction are formed via endochondral ossification. In this process, mesenchyme—derived anteriorly from neural crest cells and posteriorly from mesoderm/somites—first differentiates into cartilage before bone mineralization. Disturbances in mesenchymal development may result from abnormal gene signaling from the neural tube, involving the dorsalizing bone morphogenetic protein and ventralizing Sonic Hedgehog signaling cascade [7, 10, 53].

In primary clival encephalocele, the malformation originates from abnormalities in the notochordal and paraxial mesoderm, which give rise to somites and sclerotomes. These structures form the basioccipital and posterior sphenoid bones. The clivus, an endochondral bone, undergoes chondrification, followed by cartilage resorption and subsequent bone deposition. This process starts from the basioccipital to the sphenoid portion [42]. At birth, both regions are partially ossified and separated by the spheno-occipital synchondrosis. Failures in cartilage resorption can result in variations of the clivus [32, 42]. To date, our literature review has identified some cases of clival meningocele associated with other craniocervical junction malformations [19, 24, 28, 41, 46]. The craniocervical junction itself develops from four occipital somites and the first three cervical somites.

Congenital clival defects may arise from cellular and extracellular disturbances, including genetic or vascularization abnormalities, infections, exposure to radiation, and drugs. Amniotic band syndrome, also known as constriction ring syndrome, occurs when fibrous bands of the amniotic sac entangle developing fetal structures. Another rare etiology of clival meningocele involves defects in midline integration at various stages of embryogenesis [28]. Several theories have been proposed to explain this malformation, such as failed twinning, teratogenic insults, splitting of the notochord or prechordal plate cells, and variants of the median cleft face syndrome [28, 47].

Some rare occult bony defects can be found in the clivus. One anomaly is the CBM, a small, well-defined canal that originates on the intracranial surface of the clivus, typically involving the basioccipital portion [8]. Bony canals traverse the clivus, which contain communicating vessels with the internal and external venous base. The CBM may have a vascular origin, due to emissary veins, or may result from a canal formation along notochordal remnants. Due to its small size, the CBM is usually not related to meningoceles. However, in a minority, high CSF pulse pressure can enlarge the bony opening canals, potentially leading to the protrusion of nervous tissue [38]. As rare cases of CBM-associated meningoceles result from elevated CSF pressure, they were excluded from this analysis.

Even when clival meningoceles are classified as primary and associated with anatomical failures, it is sometimes impossible to completely rule out the influence of multifactorial and acquired factors that could facilitate the herniation of the dura mater, arachnoid, CSF, and brain tissue. These factors are not as apparent as trauma or intracranial hypertension. Examples include excessive pneumatization of the sphenoid bone, the pulsatile effects of nearby arteries, and CSF pressure pulses that may contribute to the development of small osseous defects in the clivus. Intracranial pressure fluctuates with the cardiac cycle, influenced by arterial, venous, and CSF flow dynamics and cerebral compliance. CSF movements may induce gradual meningeal herniation through the skull [49].

The clinical presentation of clival meningocele in children includes respiratory insufficiency due to airway obstruction, CSF fistula, and recurrent episodes of meningitis [3, 19, 36, 38, 40, 41]. An atypical case of a large clival meningocele presented with neck swelling in an adolescent [46]. In adults, the clinical course is more insidious, often with rhinorrhea and bacterial meningitis [1, 23, 27, 52, 54]. Late manifestations may result not only from congenital factors contributing to clival failure but also from acquired mechanisms that have not yet been identified [23, 54]. For diagnosis, CT scans are effective in evaluating bone structures, while the higher contrast resolution of MRI scans helps in identifying the location of clival lesions [11]. Advanced imaging techniques such as angiography or tractography were not mentioned but are likely reasonable options for selected cases. Since symptoms vary between patients and malformations can coexist, further testing may be indicated individually according to the physician’s evaluation.

When classifying clival meningoceles, it is essential to consider the clivus as an anatomical landmark. In all cases, the location of the bony defect (posterior transsphenoidal, spheno-occipital, or basioccipital) was the most important factor in guiding the choice of surgical route. The endoscopic endonasal transsphenoidal approach (EETA) appears more favorable for transsphenoidal meningocele, likely due to the proximity of the posterior nasal cavity to the upper clivus. The EETA also offers a panoramic view of the surgical field and enables the use of a vascularized nasoseptal flap [21]. Although earlier reports did not specify the type of intraoperative magnification used, it is reasonable to assume that microscopes were employed up to the 1980s and 1990s, whereas the endoscope likely became the chosen method from the 2000s onward [6]. For basioccipital meningoceles, the transoral approach via endoscopy, microsurgery, or direct view may be more appropriate. It provides optimal surgical exposure, a direct trajectory to the lower clivus, and is considered a safe and effective treatment option. The transoral route can be satisfactorily performed even in neonates with small oral cavities [38].

The length of the bony defect did not significantly influence the choice between transoral or endonasal approaches. Most defects were relatively small, ranging from 5 to 20 mm. This may reflect selection bias, as large congenital skull base defects could be incompatible with life. Only one patient presented with a significantly large bony defect; in this case, surgical repair was not feasible, and a thecoperitoneal shunt was placed instead [46].

Other anatomical structures surrounding the clivus include the soft and hard palate and cleft palate. In a standard manner, the soft palate was retracted superiorly toward the nasal cavity to allow transoral access. No cases of cleft palate were identified. Notably, neither the soft palate nor the hard palate interfered with the surgical approach.

An important concern is the skull base repair following surgical procedures, as postoperative CSF leak is a significant source of morbidity and can result in life-threatening intracranial infections. The introduction of the nasoseptal flap in 2006 revolutionized endoscopic endonasal skull base surgery and facilitated the expansion of this treatment modality [14, 15].

Because the nasoseptal flap cannot be harvested via the transoral approach, some authors have employed direct suturing and multilayer reconstruction techniques using fat grafts, fascia lata, collagen, and fibrin glue [3, 36, 38]. This route also allows direct repair of the dural sac with suture, either through endoscopic or direct view [38]. Given the diversity of closure techniques and their favorable surgical outcomes—regarding postoperative CSF leak and meningitis— we cannot determine the best method at this time.

In our illustrative case, initial care at a small hospital with limited resources resulted in delayed specialist evaluation and imaging. The patient was referred to a larger hospital for definitive management after authorization from the inter-hospital transfer regulatory system. The transoral approach was selected over an endonasal route due to the proximity of the basioccipital meningocele to the lower third of the clivus. This corridor allowed complete resection of the meningocele sac and direct dural repair with 4–0 polypropylene suture, followed by an abdominal fat graft and fibrin glue placement. Given the neurosurgical team’s experience with neuroendoscopic procedures, including intraventricular and skull base surgery, this method was preferred over traditional open approaches (direct view). After careful literature review, we noted that this is the first reported endoscopic transoral repair of a clival meningocele. Our case demonstrates a successful surgical outcome. Panagopoulos et al. [38] previously described the same route, although using direct view rather than an endoscopic approach.

This study has some limitations. Regarding the systematic review, the overall quality of the included studies ranged from low to moderate, reflecting methodological limitations such as lack of follow-up, publication bias, and limited standardization of these study designs. However, given the rarity of primary clival meningoceles and the absence of cohort studies, case–control studies, and randomized controlled trials, the quality assessment can be considered moderate to acceptable. To date, no classification system for clival meningoceles has been described. Although the present proposal was developed to assist in the selection of the most appropriate surgical approach for each type, it was based on a small number of reported cases and heterogeneous clinical data. Therefore, it cannot be considered clinically validated without further studies. The anatomical study is limited by its small sample size, qualitative nature, and reliance on cadaveric dissections, which may not reflect population variability or fully replicate clinical conditions associated with clival meningocele anatomy. Finally, the case report is naturally limited in generalizability, as it represents the clinical and surgical course of a single patient.

## Data Availability

The author confirms that all data generated or analysed during this study are included in this published article.
